# Mass Spectrometry Analysis and Biological Characterization of the Predatory Ant *Odontomachus monticola* Venom and Venom Sac Components

**DOI:** 10.3390/toxins11010050

**Published:** 2019-01-17

**Authors:** Naoki Tani, Kohei Kazuma, Yukio Ohtsuka, Yasushi Shigeri, Keiichi Masuko, Katsuhiro Konno, Hidetoshi Inagaki

**Affiliations:** 1 Liaison Laboratory Research Promotion Center, Institute of Molecular Embryology and Genetics, Kumamoto University, 2-2-1 Honjo, Chuo-ku, Kumamoto 860-0811, Japan; naotani@kumamoto-u.ac.jp; 2 Eco-Frontier Center of Medicinal Resources, School of Pharmacy, Kumamoto University, 5-1 Oe, Chuo-ku, Kumamoto 862-0973, Japan; cokazuma@kumamoto-u.ac.jp; 3 Biomedical Research Institute, National Institute of Advanced Industrial Science and Technology (AIST), 1-1-1 Higashi, Tsukuba, Ibaraki 305-8566, Japan; y-ohtsuka@aist.go.jp; 4 Department of Chemistry, Wakayama Medical University, 580 Mikazura, Wakayama 641-0011, Japan; yshigeri@wakayama-med.ac.jp; 5 School of Business Administration, Senshu University, 2-1-1 Higashimita, Tama-ku, Kawasaki 214-8580, Japan; kmasuko@isc.senshu-u.ac.jp; 6 Institute of Natural Medicine, University of Toyama, 2630 Sugitani, Toyama, Toyama 930-0194, Japan; kkgon@inm.u-toyama.ac.jp

**Keywords:** ant, venom, mass spectrometry analysis, pilosulin-like peptide

## Abstract

We previously identified 92 toxin-like peptides and proteins, including pilosulin-like peptides 1–6 from the predatory ant *Odontomachus monticola*, by transcriptome analysis. Here, to further characterize venom components, we analyzed the venom and venom sac extract by ESI-MS/MS with or without trypsin digestion and reducing agent. As the low-molecular-mass components, we found amino acids (leucine/isoleucine, phenylalanine, and tryptophan) and biogenic amines (histamine and tyramine) in the venom and venom sac extract. As the higher molecular mass components, we found peptides and proteins such as pilosulin-like peptides, phospholipase A_2_s, hyaluronidase, venom dipeptidyl peptidases, conotoxin-like peptide, and icarapin-like peptide. In addition to pilosulin-like peptides 1–6, we found three novel pilosulin-like peptides that were overlooked by transcriptome analysis. Moreover, pilosulin-like peptides 1–6 were chemically synthesized, and some of them displayed antimicrobial, hemolytic, and histamine-releasing activities.

## 1. Introduction

Ants (Hymenoptera: Formicidae) have been believed to share the same ancestor with bees and wasps, and have many traits in common with them. Since most ant species have a sting with venoms including formic acid, hydrocarbons, amines, peptides, and proteins, for predatory purpose [1], the venom components have been attractive as potential lead compounds for drug development. One of the peptide components in the ant venom is the pilosulin-like peptide. Since Donovan et al. isolated pilosulin 1 cDNA from a *Myrmecia pilosula* cDNA library in 1993 [2], many pilosulin and pilosulin-like peptides have been identified from various ant species [3,4,5]. Some of the pilosulins and pilosulin-like peptides formed homo- or heterodimers by single or double disulfide bridges, and displayed various bioactivities [2,6,7].

*Odontomachus monticola*, a predatory ant species in the subfamily Ponerinae, is about 10 mm long, red-brown in color, and has long mandibles [8]. They prey on other insects using their venomous stings, which cause intense pain and prolonged itching in humans. In 2016, Aili et al. reported 528 molecular masses, including 27 disulfide-bonded, without information on their primary structures or biological activities, in the venom of *O. hastatus* by LC-MS analysis [9]. On the other hand, we identified 92 toxin-like peptides and proteins including pilosulin-like peptides 1–6 from *O. monticola* by transcriptome and peptidome analysis [10]. Despite the transcriptome and limited peptidome analysis, the venom components of the ant genus *Odontomachus* have not been fully understood.

In this study, we further analyzed the venom and venom sac extract by ESI-MS/MS with or without trypsin digestion and reducing agent. Our results uncovered 1244 molecular masses, including toxin-like peptides and toxin-like proteins in the venom and venom sac extract of *O. monticola* by ESI-MS/MS without trypsin digestion, and also confirmed the amino acid sequences, amidation, processing, and dimerization with a single disulfide bridge of pilosulin-like peptides. Furthermore, we characterized the physicochemical and biological properties of pilosulin-like peptides. Some of the pilosulin-like peptides have a cationic amphiphilic region and displayed antimicrobial, hemolytic, and histamine-releasing activities.

## 2. Results and Discussion

### 2.1. Low-Molecular-Mass Components in O. monticola Venom and Venom Sac Extract

**Figure 1:**
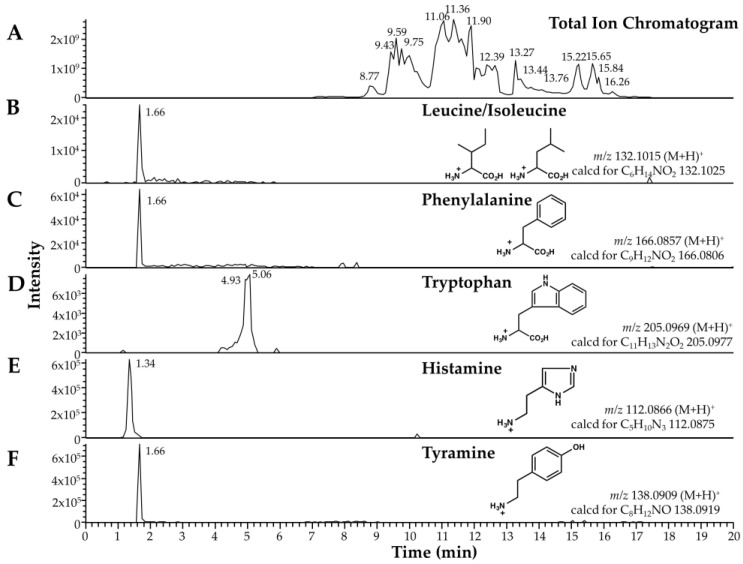
Low-molecular-mass components of *O. monticola* venom and venom sac extract as analyzed by LC-ESI-MS. (**A**) Selected ion chromatograms of total ion chromatogram, (**B**) leucine/isoleucine, (**C**) phenylalanine, (**D**) tryptophan, (**E**) histamine, and (**F**) tyramine. The observed and calculated *m/z* values are shown with the corresponding structural formulae.

Among the low-mass components, we found two biogenic amines (histamine and tyramine) and three amino acids (leucine/isoleucine, phenylalanine, and tryptophan) in the venom and venom sac extract of *O. monticola* by retention time comparison and elemental analysis (Figure 1).

Histamine and tyramine interact with their specific receptors and activate specific neurons, being major neurotransmitters in insects, to regulate physiology and behavior. In addition, histamine is partly involved in pain-producing and itching reactions [11]. According to the early studies about ant venoms, histamine has been detected in several ant species [12]. In *Myrmecia pyriformis*, histamine accounted for approximately 2% of venom dry weight [13]. Histamine might be a major and common biogenic amine in ant venoms. Glutamic acid, a neurotransmitter and neurotoxin, was a major and common component in the venoms of five ant species, *Myrmica ruginodis*, *Pogonomyrmex badins*, *Solenopsis saevissima*, *Tetramorium guineense*, and *T. caespitum* L [12], but did not exist in the venom and venom sac extract of *O. monticola*. Most of the ant species might have biogenic amines and amino acids in various combinations. Although social and solitary wasps often have nucleosides (e.g., adenosine and guanosine) and nucleotides (e.g., AMP and ADP), this ant species lacks these compounds in its venom [14].

### 2.2. Overview of High-Molecular-Mass Components in Venom and Venom Sac Extract

We determined the amino acid sequences from ESI-MS/MS data and searched the amino acid sequences using PEAKS 8.5 against the major 192 components from *O. monticola* venom gland transcriptome data (Table S1). These components were selected from the transcripts which have high relative expression level in the transcriptome analysis or have some similarities with the transcripts of well-known toxins. MS/MS analysis under reducing and nonreducing conditions without trypsin digestion yielded 973 and 517 amino acid sequences (247 overwrapped sequences), respectively. Overall, 545 sequences from a total of 1244 sequences were derived from pilosulin-like peptides.

From the 1244 sequences, 193 were mapped to 50 high-molecular-mass components other than pilosulin-like peptides. The amino acid sequences from MS/MS data without trypsin digestion suggests that some of the proteins might be partially degraded before or after the extraction step. Due to the limited number of sequences in the reference database of the PEAKS 8.5 software, we considered that many of the de novo amino acid sequences were not assigned. Next, we searched the de novo amino acid sequences against 41,764 contig sequences of transcriptome analysis [10] with the tbalstn program to prevent oversight. In this way, 109 sequences were additionally mapped to 70 high-molecular-mass components other than pilosulin-like peptides (Table S2), and 397 sequences were unassigned. Including the MS/MS data with trypsin digestion with the data without trypsin digestion, the amino acid sequences were mapped to 104 components other than pilosulin-like peptides in the venom and venom sac extract by PEAKS 8.5 (Table 1). The total number of components identified from mass spectrometry analysis (183) is much smaller than that of contigs yielded from transcriptome analysis (41,764).

Collecting the venom by electrostimulation has been successful in some large ant species [5]. Since most ant species are too small to apply electrostimulation for collecting venom, a method of dissecting and extracting the venom sac has been applied to collect venoms in many ant species. Because we collected the venom from dissected venom sacs, membrane proteins, cytoskeleton proteins, DNA-binding proteins, translation-related proteins, and replication-related proteins are not believed to represent venom components and are most likely contaminants from venom sac tissue.

The rest of the components were roughly classified into three groups: toxin-like components, non-toxin-like components, and uncharacterized components. Among the toxin-like components we found pilosulin-like peptides, phospholipase A_2_s, hyaluronidase, and venom dipeptidyl peptidases of which amino acid sequences have some similarities to those of *Apis mellifera* orthologues in our previous study [10]. These proteins might function in tissue damage, venom diffusion, and venom maturation. Nontoxin-like components included cytochrome P450s and pheromone-binding proteins. 

According to the classification of the venom components in *Solenopsis invicta* [15], these proteins might be classified into the groups of self-venom protection and chemical communication for alarm. Among uncharacterized components, we found uncharacterized proteins 1, 3, and 4. Uncharacterized protein 3 has nine cysteine residues, like some of the well-characterized toxins which are rich in cysteine residues, allowing them form unique conformations [16,17].

### 2.3. Novel Pilosulin-Like Peptides

**Figure 2:**
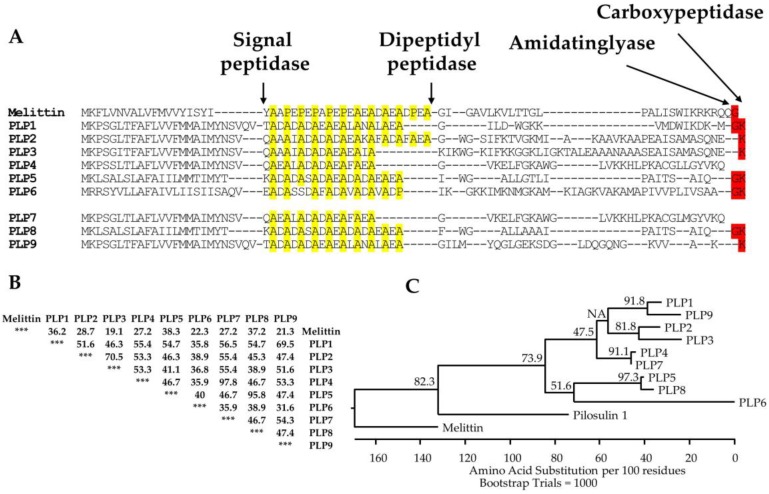
Multiple alignment, identity matrix, and phylogenic analysis of pilosulin-like peptides. (**A**) The amino acid sequences of melittin and pilosulin-like peptides were aligned with ClustalW in Lasergene 12 (DNASTAR, Madison, WI, USA) and manually modified. Arrows indicate the putative processing and modification sites for signal peptidase, dipeptidyl peptidase, amidatinglyase, and carboxypeptidase. Proline and alanine residues in the spacer region between the signal and mature peptides of pilosulin-related peptides are highlighted in yellow. Nucleotide sequences for pilosulin-like peptides 7, 8, and 9 were assigned DDBJ/EMBL/GenBank Accession Numbers LC416796–LC416798, respectively. (**B**) Percentage amino acid sequence identities between melittin and pilosulin-like peptides are shown. (**C**) The alignment of pilosulin-like peptides, pilosulin 1, and melittin precursors by ClustalV in Lasergene 12 was used to construct a phylogenic tree using the neighbor-joining (NJ) method. The phylogenic tree rooted with the amino acid sequence of melittin. The numbers above the branches indicate the percentage of 1000 bootstrap replicates.

MS/MS analysis under reducing and nonreducing conditions without trypsin digestion showed that 545 sequences of 1244 total sequences were derived from pilosulin-like peptides; pilosulin-like peptides 1, 2, 3, 4, 5, and 6 have 47, 124, 98, 82, 34, and 101 derivatives, respectively. They included the precursors and degradation products (Table 2; Figure 2B; Figures S1–S6). Because pilosulin-like peptides 2 and 3 have closely related amino acid sequences, they share five common derivatives (Table 2; Figures S2 and S3). Furthermore, we found three novel pilosulin-like peptides in the de novo amino acid sequences, which we termed pilosulin-like peptides 7, 8, and 9 (Figure 2A). To isolate pilosulin-like peptides 7 and 8 cDNAs, we revised raw reads of the transcriptome data in our previous study and found several reads corresponding to pilosulin-like peptides 7 and 8. cDNA clones of pilosulin-like peptides 7 and 8 were isolated by RT-PCR (Figures S10 and S11), indicating that pilosulin-like peptides 4 and 7 and pilosulin-like peptides 5 and 8 each have related nucleotide sequences. Accordingly, four nucleotide sequences (pilosulin-like peptides 4, 5, 7, and 8) were integrated into two sequences (pilosulin-like peptides 4 and 5) by the cd-hit-est software during the assembly process of transcriptome analysis [10]. Pilosulin-like peptides 7 and 8 have 83 and 27 derivatives in the 1244 sequences, respectively. Pilosulin-like peptides 4 and 7 and pilosulin-like peptides 5 and 8 share 41 and 5 common derivatives, respectively (Table 2; Figure 2B; Figures S7 and S8). 

We found pilosulin-like peptide 9 in the annotation of de novo amino acid sequences. The unassigned de novo amino acid sequences of MS/MS analysis with trypsin digestion were compared against 41,764 contig sequences of transcriptome analysis [10] by the tblastn program. One of the amino acid sequences (Met-Tyr-Gln-Gly-Leu-Gly-Glu-Lys) (Figure S9) was matched to the contig Om11177_c0_g1_i2, which was annotated to aspartyl/glutamyl tRNA in our previous study [10]. Although tRNAs must be eliminated during the extraction step of total RNA and must not be transcribed by reverse transcriptase, the relative expression level of Om11177_c0_g1_i2 was high and accounted for 1.3% of all reads; so, we considered the contig Om11177_c0_g1_i2 as being a misassembled product of the Trinity software. 

We selected the raw reads that encoded the amino acid sequence Met-Tyr-Gln-Gly-Leu-Gly-Glu-Lys and manually assembled the selected reads. The assembled nucleotide sequence encoded a similar leader sequence with pilosulin-like peptides 1–8, and we considered the manually assembled contig as encoding a novel member of the pilosulin-like peptide family. Using the contig sequence, we designed oligonucleotide primers and isolated a DNA fragment encoding the entire open reading frame (ORF) of pilosulin-like peptide 9 by RT-PCR (Figure S12).

Interestingly, the cDNA of pilosulin-like peptide 9 had a structure in which a nucleotide sequence (5′-ATGTACCAAG-3′) was inserted between the nucleotide positions 214 and 215 of the pilosulin-like peptide 1 cDNA. As the result of insertion and frame shifting, the downstream amino acid sequence of pilosulin-like peptide 9 is far different from that of pilosulin-like peptide 1. This may indicate the process of diversification of pilosulin-like peptides. Although we predicted an amino acid sequence of the mature peptide from the nucleotide sequence, only a partial amino acid sequence of pilosulin-like peptide 9 has been confirmed by MS/MS analysis.

### 2.4. Mature Forms of Pilosulin-Like Peptides

**Figure 3:**
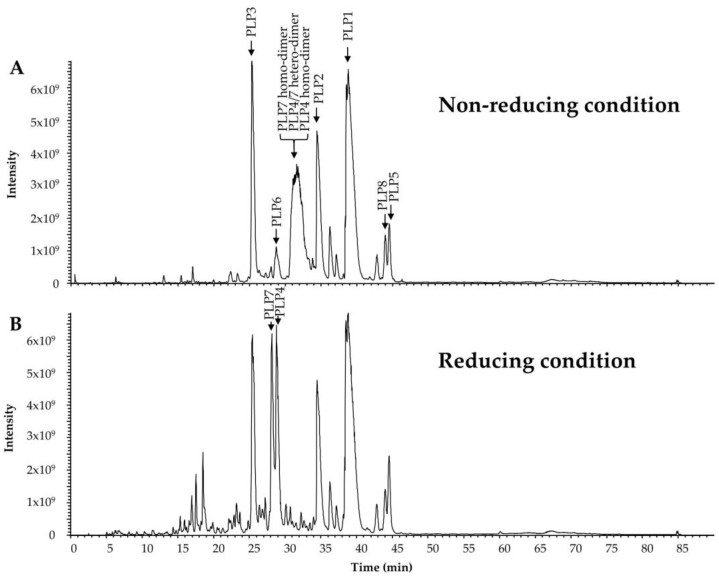
High-molecular-mass components of *O. monticola* venom and venom sac extract as analyzed by LC-ESI-MS. (**A**) The patterns of the total ion current of *O. monticola* venom and venom sac extract under nonreducing and (**B**) reducing conditions are shown. Peaks containing pilosulin-like peptides are labeled by arrows.

**Figure 4:**
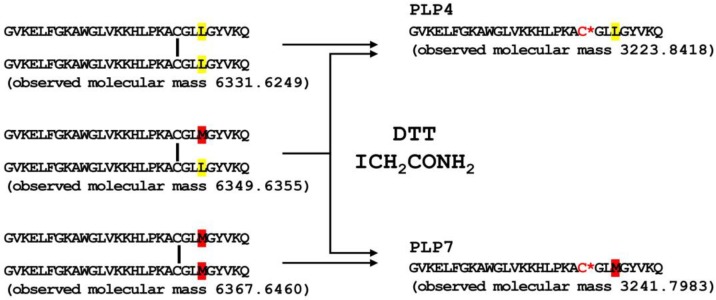
Dimer formation of pilosulin-like peptides 4 and 7. Monomers of pilosulin-like peptides 4 and 7 were connected by a disulfide bridge at the amino acid position 21. Unique amino acid residues in pilosulin-like peptides 4 and 7 are highlighted in yellow and red, respectively. C* indicates S-(carbamoylmethyl)-L-cysteine.

In our previous study, we predicted the N-termini of every mature pilosulin-like peptide, the elimination of Lys residues at the C-termini, and the amidation at the C-termini of some of the mature pilosulin-like peptides [10]. Signal peptidase, dipeptidyl peptidase, amidatinglyase, and carboxypeptidase, some of which are detected by ESI-MS/MS analysis (Table 1 and Table S2), might be involved in the processing and modification of pilosulin-like peptides (Figure 2A). The peptides of highest or second-highest signal intensity corresponded to the predicted mature peptides by nucleotide sequences. Since the signal intensity is affected by the abundance of the peptide in the venom and venom sac extract, the peptides of highest or second-highest signal intensity might be the major components in the venom. In addition to the mature peptides, we found the precursors of pilosulin-like peptides that have Gly or Gly–Lys residues at the C-termini and the degradation products of pilosulin-like peptides (Table 2). We compared the LC-ESI-MS profile of the crude venom and venom sac extract under reducing and nonreducing conditions. A broad peak (retention time around 31.58 min), which consisted of three masses (6331.6249, 6349.6355, and 6367.6460) under the nonreducing condition, was divided into two peaks (retention time 28.14 and 28.80 min), which consisted of two masses (3223.8418 and 3241.7983) under the reducing condition (Figure 3). These observed molecular masses were identical to the theoretical masses of mature pilosulin-like peptides 4 and 7 monomers, respectively. Consequently, we confirmed that pilosulin-like peptides 4 and 7 formed a homo- or heterodimer by a disulfide bridge (Figure 4).

### 2.5. Structural and Physicochemical Properties of Pilosulin-Like Peptides

**Figure 5:**
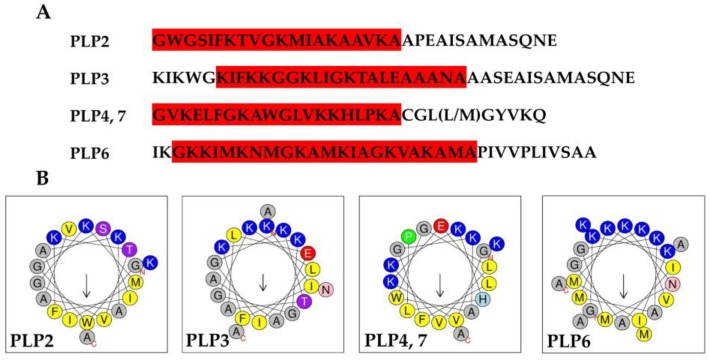
Amino acid sequences and helical wheel projection of pilosulin-like peptides 2, 3, 4, 6, and 7. (**A**) Amino acid sequences of mature pilosulin-like peptides. The cationic amphipathic helix regions in pilosulin-like peptides (PLP) 2, 3, 4, 6, and 7 predicted by HeliQuest are highlighted in red. (**B**) Helical wheel projections of pilosulin-like peptides 2, 3, 4, 6, and 7 drawn by HeliQuest. Nonpolar amino acids (F, I, L, M, V, and W), basic amino acids (K), acidic amino acids (D), small polar amino acids (A and G), aromatic polar amino acids (H), hydroxyl-containing polar amino acids (S and T), amide-containing polar amino acids (N), and proline (P) are highlighted by yellow, blue, red, gray, sky-blue, purple, pink, and green color, respectively.

Pilosulins are α-helical cationic antimicrobial peptides [2,6,7]. We examined the secondary structure of pilosulin-like peptides using Proteus [18]. Proteus showed that mature regions of pilosulin-like peptides 1–8 are α-helical structures, but pilosulin-like peptide 9 is a coiled-coil structure. The pI of the mature pilosulin-like peptides, without consideration of amidation at C-termini, was calculated with IPC [19]. IPC demonstrated that the mature pilosulin-like peptides 5 and 8 are acidic peptides (calculated pI = 5.98) and the others are basic peptides. The calculated pIs of the mature pilosulin-like peptides 1, 2, 3, 4, 6, 7, and 9 are 8.46, 8.89, 9.06, 9.18, 9.96, 9.18, and 8.46, respectively. Taken together, pilosulin-like peptides 1–4, 6, and 7 may function as α-helical cationic antimicrobial peptides. To test whether pilosulin-like peptides have a cationic, amphiphilic helical conformation, the net charge and hydrophobic indexes of pilosulin-like peptides were examined using HeliQuest [20]. Parameters to search cationic amphipathic α-helix regions of pilosulin-like peptides were determined based on a well-known cationic amphipathic antimicrobial peptide, cecropin A (GenBank accession No. AAA29185): hydrophobicity 0–0.6, hydrophobic moment 0.1–1.0, and net charge 3–10. HeliQuest found cationic amphipathic α-helix sequences in pilosulin-like peptides 2–4, 6, and 7 (Figure 5A). Furthermore, helical wheel projections demonstrated that they are typical cationic α-helical amphiphilic peptides, in which hydrophobic amino acids are located on one side and basic amino acids are on the other side (Figure 5B). However, HeliQuest could not find an α-helical region that showed a typical cationic amphiphilic peptide in pilosulin-like peptide 1, since the mature form of pilosulin-like peptide 1 is shorter than the default setting (18 amino acids) of HeliQuest and the net charge is less than two.

### 2.6. Biological Activities of Pilosulin-Like Peptides

Pilosulin-like peptides 1–6 were chemically synthesized by the 9-fluorenylmethyloxycarbonyl (Fmoc) method, and we examined their biological activities (Table 3). We synthesized homodimeric pilosulin-like peptide 4, which was linked by a single disulfide bond. Pilosulin-like peptides 1–4 and 6 with a cationic α-helix displayed antimicrobial activities against *Escherichia coli* and *Staphylococcus aureus*. These activities were higher than those of magainin, a well-known *Xenopus laevis* antimicrobial peptide [21]. Interestingly, pilosulin-like peptide 4 also had high antimicrobial activities against *Saccharomyces cerevisiae*. In support of these experimental results, the antifungal peptide prediction server Antifp predicted that pilosulin-like peptide 4, but not other pilosulin-like peptides, was an antifungal peptide [22]. Virtual alanine scanning of pilosulin-like peptide 4 by Antifp revealed that the replacement of a cysteine residue remarkably reduced the index of antifungal activity. Cysteine residues are known to be abundant in antifungal peptides in general [22], and the replacement of cysteine with serine in brevinin-1B Ya, a frog antimicrobial peptide, reduced the antifungal activity [23]. The cysteine residue of pilosulin-like peptide 4 may be important for antifungal activity.

Pilosulin-like peptide 5 with an acidic α-helix had no or low antimicrobial activities against all the microbes tested in this study, but had the highest hemolytic activity among pilosulin-like peptides 1–6. This hemolytic activity may depend on higher hydrophobicity. Previous studies suggested a correlation between peptide hydrophobicity and hemolytic activity [25]. For example, melittin, a honey bee hemolytic peptide, shows a higher hydrophobicity (0.45–0.88). Hydrophobicity of pilosulin-like peptide 5 is 0.914, which is the highest among pilosulin-like peptides.

Pilosulin-like peptides are major components of *O. monticola* venom sac extract. The first step in the antimicrobial mechanism has been suggested to be these peptides binding to the anionic phospholipids that are abundant in bacterial membranes, which is then followed by pore formation by α-helical cationic peptides as the second step [26]. The variations of antimicrobial activities might reflect on the difference of the physicochemical properties among pilosulin-like peptides.

Pilosulin-like peptide 4 had histamine-releasing activity against rat mast cells comparable to that of melittin, which displays histamine-releasing activity.

## 3. Conclusions

In this study, we have analyzed the venom and venom sac extract of *O. monticola* by mass spectrometry analysis. Because of the high sensitivity of the analysis, the extract accounting for just one fifth of a venom sac was enough for a single MS/MS analysis. We determined 1244 amino acid sequences by ESI-MS/MS analysis without trypsin digestion. In total, 545 of them were derived from pilosulin-like peptides 1–8, and 302 amino acid sequences corresponded to the high molecular mass components that were identified from our previous transcriptome analysis. This MS/MS analysis suggests that the majority of the venom components might be 2–6.5-kDa peptides.

Synthetic pilosulin-like peptides 1–4 and 6 displayed antimicrobial and histamine-releasing activities. Moreover, pilosulin-like peptide 5 showed the highest hemolytic activity among pilosulin-like peptides. Some of the ant toxins show various biological activities, such as neurotoxic [27,28] and enzymatic activity [29]. In future studies, we will further explore the multiple activities of pilosulin-like peptides.

## 4. Materials and Methods

### 4.1. Ants

One *O. monticola* colony was collected in Musashimurayama, Tokyo, Japan, on 1 July 2016. The species was morphologically identified.

### 4.2. Liquid Chromatography-Mass Spectrometry (LC-MS) Analysis for Low-Molecular-Weight Components

Twenty *O. monticola* venom sacs were collected and extracted with 50% acetonitrile containing 0.1% (*v*/*v*) trifluoroacetic acid (50 μL) for 2 h at 4 °C. A single venom sac contains 50–150 nL of venom. The extract was passed through a 0.45-μm filter and successively diluted with the extraction solvent to a final concentration of 0.04 sacs/μL. This dilution was used for the LC-MS analysis. The LC conditions were: solvent A, 0.1% (*v*/*v*) aqueous formic acid; solvent B, 0.1% (*v*/*v*) formic acid in acetonitrile; 5–65% linear gradient of solvent B in solvent A at a flow rate of 200 μL/min; column, Capcell Pak C18 UG 120 (1.5 × 150 mm, Shiseido, Tokyo, Japan); column temperature, 25 °C. The molecular weights of the ant peptides were verified by LTQ Orbitrap XL-ETD (Thermo Fisher Scientific, Waltham, MA, USA). The MS conditions were: ionization, electrospray in positive mode; ion spray voltage, 4.6 kV; capillary temperature, 350 °C; capillary and tube lens voltages, 19 V and 35 V, respectively; detector, an Orbitrap at a resolution of 60,000 at *m*/*z* 400. MS scan range was *m*/*z* 100–2000. The mass spectrometer was calibrated with polytyrosine, and the resolution was usually 1–3 ppm after measurement.

### 4.3. Liquid Chromatography-Mass Spectrometry (LC-MS) Analysis for Peptide and Protein Components

After filtration of the venom sac extract, the extract was diluted 10 times with 50 mM ammonium bicarbonate, pH 8.0, to improve separation and prevent peak tailing in HPLC. The diluted extract was reduced with DTT at a concentration of 10 mM (Thermo Fisher Scientific), alkylated with iodoacetamide at a concentration of 20 mM (Thermo Fisher Scientific), or digested by trypsin at a concentration of ca. 30 μg/mL (Promega, Madison, WI, USA) overnight at 37 °C. Separation was achieved by Zaplous α pep C18 analytical column (3 μm 120A, 1.5 × 150 mm, AMR, Tokyo, Japan) with L-Trap column (5 μm, 0.3 × 5 mm, AMR, Tokyo, Japan) at a flow rate of 500 μL/min using two mobile phases, 0.1% (*v*/*v*) aqueous formic acid (solvent A) and 0.1% (*v*/*v*) formic acid in acetonitrile (solvent B). The following gradient was used: 0–60 min, 5–65% solvent B; 60–70 min, 65–95% solvent B; 70–80 min, 95% solvent B.

The molecular weights of the ant peptides were verified by Q Exactive (Thermo Fisher Scientific, Waltham, MA, USA). The MS conditions were: ionization, nanoelectrospray (CaptiveSpray Ionization: CSI) in positive mode; ion spray voltage, 1.4 kV; capillary temperature, 250 °C; S-lens level, 50; detector, an Orbitrap at a resolution of 70,000 from *m*/*z* 350–2000. The mass spectrometer was calibrated with a calibrant of LTQ Velos ESI Positive Ion Calibration Solution (Thermo Fisher Scientific), and the mass accuracy was usually 1–3 ppm after measurement. The raw mass spectrum was processed by using Xcalibur (Thermo Fisher Scientific). Peptide sequences were determined from MS/MS spectra by PEAKS 8.5 (Bioinformatics Solutions, Waterloo, Canada; parent mass error tolerance 10.0 ppm, fragment mass error tolerance 0.02 Da, score threshold 15.0) and manually checked. We selected 192 amino acid sequences derived from major transcripts in the venom gland transcriptome analysis and the 116 amino acid sequences of common external contaminants from cRAP (Global Proteome Machine Organization). After construction of a FASTA format file including 308 amino acid sequences, the file was incorporated into PEAKS 8.5 software as the reference database.

To assign the de novo amino acid sequences, tblastn (parameters: *E*-value 0.1, matrix PAM40, word size 3) was performed using 41,764 contig sequences of transcriptome analysis.

### 4.4. Pilosulin-Like Peptides 7–9 cDNA Cloning and Sequencing

Total RNA isolated from the ant venom glands with sacs was reverse-transcribed to cDNA and amplified by PCR with KOD-Plus-Neo (Toyobo, Osaka, Japan). The oligonucleotide primers used for pilosulin-like peptide 7 were Pilo U1 (5′-ATGAAACCGTCGGGTATCAC-3′), corresponding to nucleotides (nt) 9–28 of pilosulin-like peptide 2; 41CPas (5′-TTGCTTTACGTATCCCAT-3′); 41CP (5′-CCAAAGCGTGTGGACTGA-3′); and Oligo-dT (5′-GAGTCGACTCGAGAA(T)17-3′). 41CP and 41CPas primers were designed from transcriptome analysis data and the amino acid sequences: Met-Gly-Tyr-Val-Lys-Gln; Lys-Ala-Cys-Gly-Leu-Met. The oligonucleotide primers used for pilosulin-like peptide 8 were 51S (5′-TATGTGTGAAAGCTCTTC-3′) and 51AS (5′-CCAATGTAATGCCAATCG-3′), designed based on the 5′ and 3′ untranslated regions predicted by transcriptome analysis. The oligonucleotide primers used for pilosulin-like peptide 9 were Pilo U1, 9CPas (5′-CCCCAGTCCTTGGTACAT-3′), 9CP (5′-ATGTACCAAGGACTGGGG-3′), and Oligo-dT. 9CP and 9CPas primers were designed from the transcriptome analysis data and the amino acid sequence: Met-Tyr-Gln-Gly-Leu-Gly-Glu-Lys. The amplified products of the cDNAs were cloned into the EcoRI site of pBluescript II SK(-) (Agilent Technologies, La Jolla, CA, USA). All inserts were sequenced using a Model 3500 Genetic Analyzer (Thermo Fisher Scientific, Waltham, MA, USA).

### 4.5. Synthesis and Biological Activities of Pilosulin-Like Peptides

Pilosulin-like peptides 1–6 were prepared using Fmoc chemistry by GenScript (Nanjing, Jiangsu, China). The peptides were purified by RP-HPLC with a preparative C18 column. The purity and molecular weight of the final peptides were verified by HPLC and MS. Antimicrobial, hemolytic, and histamine-releasing activities were measured as described previously [6].

### 4.6. WEB Server Used to Analyze the Physiochemical Properties

Secondary structure prediction was performed by Proteus (http://www.proteus2.ca/proteus/). The pI of peptides was calculated with IPC (http://isoelectric.org/index.html). The physicochemical properties were examined using HeliQuest (http://heliquest.ipmc.cnrs.fr/). The antifungal peptide prediction was carried out using the Antifp server (http://webs.iiitd.edu.in/raghava/antifp/index.html).

## Figures and Tables

**Table 1:** High-molecular-mass components in the venom and venom sac extract detected by LC-ESI-MS.

Peptide/Protein	Accession Number	Coverage (%) ^a^			
		TR ^b^−, DTT ^b^−	TR−, DTT+	TR+, DTT−	TR+, DTT+
acetylcholine esterase	FX985608			1	
apolipophorin 1	FX985561	2	4	8	7
apolipophorin 2	FX985562		10	61	29
calcium-independent phospholipase A_2_ gamma	FX985510	12		7	7
carboxypeptidase D	FX985540		5	4	4
carboxypeptidase Q	FX985538			67	26
CDV3 homolog	FX986049		8		
chymotrypsin inhibitor 1	FX986026				11
cytochrome P450 1	FX985568	10		5	4
cytochrome P450 3	FX985570			11	
cytochrome P450 4	FX985571			4	
cytochrome P450 5	FX985572		9	6	9
cytochrome P450 6	FX985573		5		2
cytosolic carboxypeptidase-like protein 5	FX985541			4	7
dishevelled homolog 3	FX986050			3	4
hyaluronidase	FX985505		5	68	47
lysosomal ProX carboxypeptidase	FX985539			3	7
matrix metalloproteinase 14	FX985528			20	18
NADPH cytochrome P450 reductase	FX985574	5		6	5
neuroblastoma suppressor of tumorigenicity 1	FX986056			7	
neuropeptide-like 1	FX986015			7	7
peptidyl-prolyl cis-trans isomerase 1	FX986028			22	17
peptidyl-prolyl cis-trans isomerase 2	FX986029			4	
peptidyl-prolyl cis-trans isomerase 3	FX986030			3	8
peptidyl-prolyl cis-trans isomerase 4	FX986031			7	8
peptidyl-prolyl cis-trans isomerase 5	FX986032			6	7
peptidyl-prolyl cis-trans isomerase 7	FX986034			1	
peptidyl-prolyl cis-trans isomerase 8	FX986035	38			
pheromone binding protein 1	FX985594				14
pheromone binding protein 2	FX985595			11	
pheromone binding protein 3	FX985596			20	
pheromone binding protein 4	FX985597			16	
pheromone binding protein 6	FX985599		4		
phospholipase A_2_ isozyme 2	FX985507		11	36	40
protein disulfide isomerase 1	FX985548		8	14	8
protein disulfide isomerase 2	FX985549	19		8	
royal jelly protein	FX986021				9
UDP glucuronosyltransferase 2C1	FX986048			14	4
uncharacterized protein 1	FX985636	4	5	3	6
uncharacterized protein 3	FX986042			19	64
uncharacterized protein 4	FX986043			54	64
UPF0518 protein	FX986054	4	3	3	2
VEGF ^C^-like protein	FX985521			6	
venom allergen 1	FX985511		7		
venom allergen 2	FX985512			5	
venom allergen 3	FX985513			21	5
venom dipeptidyl peptidase 1	FX985542			4	12
venom dipeptidyl peptidase 2	FX985543			4	4
venom dipeptidyl peptidase 3	FX985544		10	3	5
venom serine carboxypeptidase	FX985537			3	
venom serine protease 2	FX985523		5		
venom serine protease 3	FX985524			6	
very high density protein 1	FX985566	3	3	3	4
very high density protein 2	FX985567		3	5	
vitellogenin 1	FX985563	3	5	5	2
vitellogenin 2	FX985564			10	
waprin 1	FX985515			22	
waprin 2	FX985516			7	11
40S ribosomal protein SA *	FX985616	8			8
60S acidic ribosomal protein P0 *	FX985621	22	6		3
60S ribosomal protein L10 *	FX985634				11
60S ribosomal protein L3 *	FX985633		10		7
60S ribosomal protein L34 *	FX985627			16	
60S ribosomal protein L36 *	FX985629			15	
60S ribosomal protein L4 *	FX985618			5	8
60S ribosomal protein L6 *	FX985625			5	11
60S ribosomal protein L7 *	FX985622				12
60S ribosomal protein L7a *	FX985617				13
60S ribosomal protein L9 *	FX985635				10
actin, muscle *	FX985587		13	7	7
ATPase WRNIP1 *	FX986047			13	
elongation factor 1-alpha *	FX985554	10		6	
elongation factor 1-beta *	FX985557		5		
elongation factor 1-delta *	FX985559			8	29
elongation factor 1-gamma *	FX985555	20		8	2
elongation factor 2 *	FX985553	4	4	2	8
elongation factor G, mitochondrial *	FX985558		2	11	
elongation factor Tu, mitochondrial *	FX985556		10		3
histone H2A *	FX985611			21	
histone H3 *	FX985610				15
laminin subunit alpha 1 *	FX985614	4	6	5	8
laminin subunit beta 1 *	FX985613		3	3	9
laminin subunit gamma 1 *	FX985612			3	3
myosin heavy chain, muscle *	FX985575	4	3	11	8
myosin heavy chain, nonmuscle *	FX985578		2	1	4
myosin IB *	FX985586			11	13
myosin Ie *	FX985583	5	7	5	9
myosin regulatory light chain *	FX985576				5
myosin Va *	FX985582	2	5	4	8
myosin VIIa *	FX985580		5	6	5
myosin XV *	FX985581	5	5	3	6
myosin XVIIIa *	FX985584	2	5	4	3
resistance to inhibitors of cholinesterase 3 *	FX986036			5	
transcription factor A, mitochondrial *	FX985588				12
transmembrane protein 214A *	FX986055		10	4	4
TRPA channel ^d,^ *	FX985591	5			
TRPM channel ^d,^ *	FX985592	10	2	5	5
TRPV channel ^d,^ *	FX985593				3
voltage-gated potassium channel Shaker *	FX985601		9		
voltage-gated sodium channel beta subunit TipE *	FX985590	9		4	2
voltage-gated sodium channel Para *	FX985589			2	10

^a^ The coverages were calculated by combining the peptide fragments with the same amino acid sequences and the same molecular masses by Peaks 8.5; ^b^ TR and DTT indicate MS/MS data with or without trypsin digestion and DTT treatment, respectively; ^c^ VEGF: Vascular Endothelial Growth Factor; ^d^ TRPA, TRPM, and TRPV channels are members of the transient receptor potential (TRP) channel superfamily; * The proteins might be derived from the venom sac.

**Table 2:** Amino acid sequences of pilosulin-like peptide derivatives analyzed from MS/MS spectra.

Toxin	Sequence	Molecular Mass	Length	Precursor Ion	RT	Intensity
PLP1	GILDWGKKVMDWIKDKMGK	2247.1907	19	750.0702	37.15	3.67 × 10^8^
	GILDWGKKVMDWIKDKMG	2119.0957	18	707.3729	38.28	9.58 × 10^9^
	GILDWGKKVMDWIKDKM-NH_2_	2061.0903	17	1031.5520	39.15	1.34 × 10^10^
	GILDWGKKVMDWIKDKM	2062.0742	17	516.5270	38.35	5.74 × 10^8^
	LDWGKKVMDWIKDKMGK	2077.0852	17	520.2803	34.11	2.65 × 10^8^
	GILDWGKKVMDWIKDK	1931.0338	16	483.7657	33.21	1.64 × 10^8^
	LDWGKKVMDWIKDKM-NH_2_	1890.9849	15	631.3347	38.44	1.79 × 10^8^
PLP2	EAGWGSIFKTVGKMIAKAAVKAAPEAISAMASQNE	3561.8323	35	891.4648	36.4	6.36 × 10^8^
	GWGSIFKTVGKMIAKAAVKAAPEAISAMASQNE	3361.7527	33	1121.5916	34.85	6.41 × 10^9^
	SIFKTVGKMIAKAAVKAAPEAISAMASQNE	3061.6304	30	766.4222	27.81	1.12 × 10^9^
	GWGSIFKTVGKMIAKAAVKAAPEAISAM	2832.5393	28	709.1410	34.52	4.53 × 10^8^
	GWGSIFKTVGKMIAKAAVKAAPEAISA	2701.4988	27	676.3826	33.09	6.43 × 10^8^
	KMIAKAAVKAAPEAISAMASQNE	2329.2134	23	777.4113	17.04	4.98 × 10^8^
	KAAVKAAPEAISAMASQNE	1885.9567	19	943.9856	15.44	9.06 × 10^8^
PLP3	KIKWGKIFKKGGKLIGKTALEAAANAAASEAISAMASQNE	4101.2407	40	1026.3179	25.25	4.47 × 10^9^
	KIFKKGGKLIGKTALEAAANAAASEAISAMASQNE	3488.8660	35	873.2313	25.16	1.83 × 10^9^
	KIKWGKIFKKGGKLIGKTALEAAANAAASEAISAM	3572.0276	35	894.0147	24.79	2.65 × 10^8^
	KKGGKLIGKTALEAAANAAASEAISAMASQNE	3100.6187	32	776.1608	25.63	2.51 × 10^8^
	KGGKLIGKTALEAAANAAASEAISAMASQNE	2972.5237	31	744.1398	27.22	2.09 × 10^8^
	GGKLIGKTALEAAANAAASEAISAMASQNE	2844.4287	30	949.1498	28.73	5.85 × 10^8^
	KTALEAAANAAASEAISAMASQNE	2319.1011	24	1160.5585	28.03	1.63 × 10^9^
PLP4	GVKELFGKAWGLVKKHLPKAC*GLLGYVKQ	3223.8418	29	806.9683	28.76	1.14 × 10^10^
	GVKELFGKAWGLVKKHLPKAC*GLL	2648.5352	24	663.1436	30.27	2.98 × 10^9^
	FGKAWGLVKKHLPKAC*GLLGYVKQ	2697.5305	24	675.3940	20.53	5.56 × 10^8^
	GKAWGLVKKHLPKAC*GLLGYVKQ	2550.4619	23	638.6231	18.8	4.34 × 10^9^
	AWGLVKKHLPKAC*GLLGYVKQ	2365.3457	21	789.4556	19.93	6.44 × 10^8^
	GKAWGLVKKHLPKAC*GLL	1975.1553	18	659.3927	17.99	6.76 × 10^8^
	KHLPKAC*GLLGYVKQ	1710.9603	15	428.7475	16	1.13 × 10^9^
PLP5	IWGALLGTLIPAITSAIQG	1894.0928	19	948.0541	43.31	3.50 × 10^8^
	IWGALLGTLIPAITSAIQ-NH_2_	1836.0873	18	919.0532	44.83	7.46 × 10^9^
	IWGALLGTLIPAITSAIQ	1837.0713	18	919.5428	42.58	3.75 × 10^7^
	ALLGTLIPAITSAIQ-NH_2_	1479.9025	15	740.9585	36.38	2.25 × 10^8^
	LLGTLIPAITSAIQ-NH_2_	1408.8654	14	705.4390	34.1	1.45 × 10^8^
	LLGTLIPAITSA	1168.7067	12	585.3602	30.26	5.15 × 10^6^
	IWGALLGTLIP	1152.6907	11	577.3530	38.86	1.34 × 10^7^
PLP6	IKGKKIMKNMGKAMKIAGKVAKAMAPIVVPLIVSAA-NH_2_	3704.2307	36	927.0673	28.61	1.58 × 10^9^
	KIMKNMGKAMKIAGKVAKAMAPIVVPLIVSAA-NH_2_	3277.9353	32	820.4910	30.81	8.46 × 10^7^
	IKGKKIMKNMGKAMKIAGKVAKAMAPIVVPL	3263.9561	31	816.9973	23.56	6.27 × 10^8^
	KNMGKAMKIAGKVAKAMAPIVVPLIVSAA-NH_2_	2905.7158	29	727.4368	30	2.21 × 10^8^
	GKAMKIAGKVAKAMAPIVVPLIVSAA-NH_2_	2532.5376	26	845.1871	29.7	1.46 × 10^8^
	AMKIAGKVAKAMAPIVVPLIVSAA-NH_2_	2347.4211	24	587.8628	31.32	1.60 × 10^8^
	KAMAPIVVPLIVSAA-NH_2_	1477.9054	15	739.9600	31.65	1.18 × 10^8^
PLP7	GVKELFGKAWGLVKKHLPKAC*GLMGYVKQ	3241.7983	29	811.4578	28.54	4.63 × 10^9^
	GVKELFGKAWGLVKKHLPKAC*GLMGY	2886.5764	26	722.6530	29.82	5.00 × 10^8^
	FGKAWGLVKKHLPKAC*GLMGYVKQ	2715.4868	24	679.8821	19.52	5.42 × 10^8^
	GVKELFGKAWGLVKKHLPKAC*GLM	2666.4917	24	667.6287	29.1	2.48 × 10^9^
	GKAWGLVKKHLPKAC*GLMGYVKQ	2568.4185	23	643.1163	17.44	4.67 × 10^9^
	LVKKHLPKAC*GLMGYVKQ	2069.1641	18	518.2991	13.87	2.37 × 10^7^
	HLPKAC*GLMGYVKQ	1600.8218	14	534.6163	16.8	9.41 × 10^8^
PLP8	FWGALLAAAIPAITSAIQG	1870.0352	19	936.0242	42.69	2.01 × 10^8^
	FWGALLAAAIPAITSAIQ-NH_2_	1812.0298	18	907.0258	44.13	2.77 × 10^9^
	GALLAAAIPAITSAIQ-NH_2_	1478.8820	16	740.4485	44.09	2.20 × 10^7^
	FWGALLAAAIPAITS	1500.8340	15	751.4250	37.82	1.60 × 10^7^
	ALLAAAIPAITSAIQ-NH_2_	1421.8606	15	711.9372	32.08	7.32 × 10^7^
	LAAAIPAITSAIQ-NH_2_	1237.7394	13	619.8778	25.83	8.77 × 10^7^
	AAAIPAITSAIQ-NH_2_	1124.6553	12	563.3350	23.08	2.19 × 10^9^

C* = S-(carbamoylmethyl)-L-cysteine. The amino acid sequences of the highest and second-highest intensities are highlighted by red and yellow, respectively.

**Table 3:** Biological properties of *O. monticola* pilosulin-like peptides.

Peptide	MIC ^a^ (μM)			Hemolytic Activity (%)		Histamine-Releasing Activity at 10 μM (%)
	*E. coli* (NBRC 14237)	*S. aureus* (NBRC 12732)	*S. cerevisiae* (NBRC 10217)	at 10 μM	at 50 μM	
PLP1	<3.1	<3.1	<50	Negative		32.9
PLP2	<6.2	<6.2	<50	Negative	10.4	30.1
PLP3	<3.1	<25	<50	Negative		37.5
PLP4	<3.1	<3.1	<3.1	Negative	10.5	66.4
PLP5	<50	Negative	Negative	6.9	94.8	28.3
PLP6	<3.1	<3.1	Negative	Negative		33.6
Magainin	<12.5	<25	Negative	-		-
Mastoparan	-	-	-	13.5 ^b^	-	31.1 ^b^
Melittin	-	-	-	100.0 ^b^	-	64.3 ^b^

^a^ MIC: minimum inhibitory concentration; ^b^ The activities indicate from Shigeri et al. [24]; -: not determined.

## Supplementary Materials

The following are available online at http://www.mdpi.com/2072-6651/11/1/50/s1, Figures S1–S9 (MS spectra of pilosulin-like peptides 1–9), Figures S9–S12 (Nucleotide and deduced amino acid sequences of pilosulin-like peptides 7–9), Table S1 (Major 192 components of *O. monticola* venom), and Table S2 (Protein assignment of the de novo amino acid sequences).
