# Supplementary material for: Mass Spectrometry Analysis and Biological Characterization of the Predatory Ant Odontomachus monticola Venom and Venom Sac Components
Source: Toxins (Basel). 2019 Jan 17;11(1):50. doi: 10.3390/toxins11010050 (PMC6356579; doi:10.3390/toxins11010050)
Supplement: Supplementary file 1 [file toxins-11-00050-s001.zip › toxins-411586-supplementary-update.pdf]

Toxins **2019**, *11*, 50; doi:10.3390/toxins11010050

Supplementary Materials: Mass Spectrometry Analysis and Biological Characterization of the Predatory Ant *Odontomachus monticola* Venom and Venom Sac Components

Naoki Tani, Kohei Kazuma, Yukio Ohtsuka, Yasushi Shigeri, Keiichi Masuko, Katsuhiro Konno and Hidetoshi Inagaki

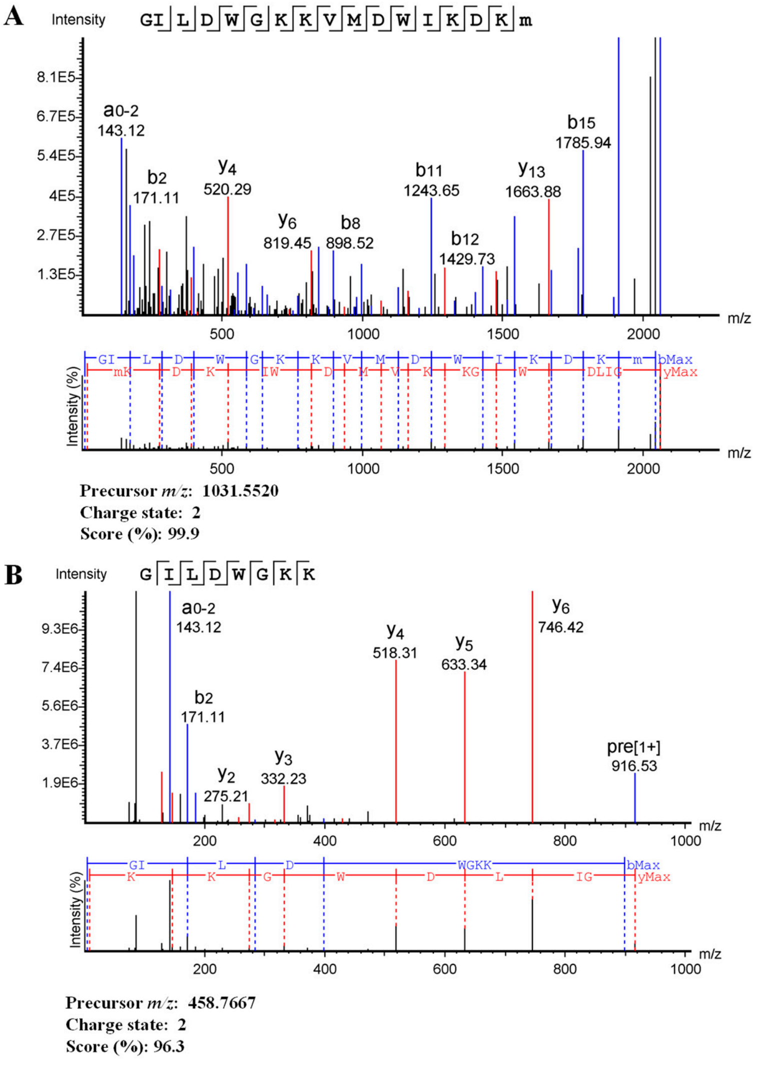

Figure S1. MS/MS spectra of pilosulin-like peptide 1. MS/MS spectra were obtained under a nonreducing condition without trypsin digestion (**A**) and under a reducing condition with trypsin digestion (**B**). The panel shows the annotated spectra and b- and y-ions of the pilosulin-like peptide 1 amino acid sequence. The peptide's precursor *m*/*z* and charge state are indicated. We referred to the transcriptome data of pilosulin-like peptide 1 for MS/MS spectrum interpretation.

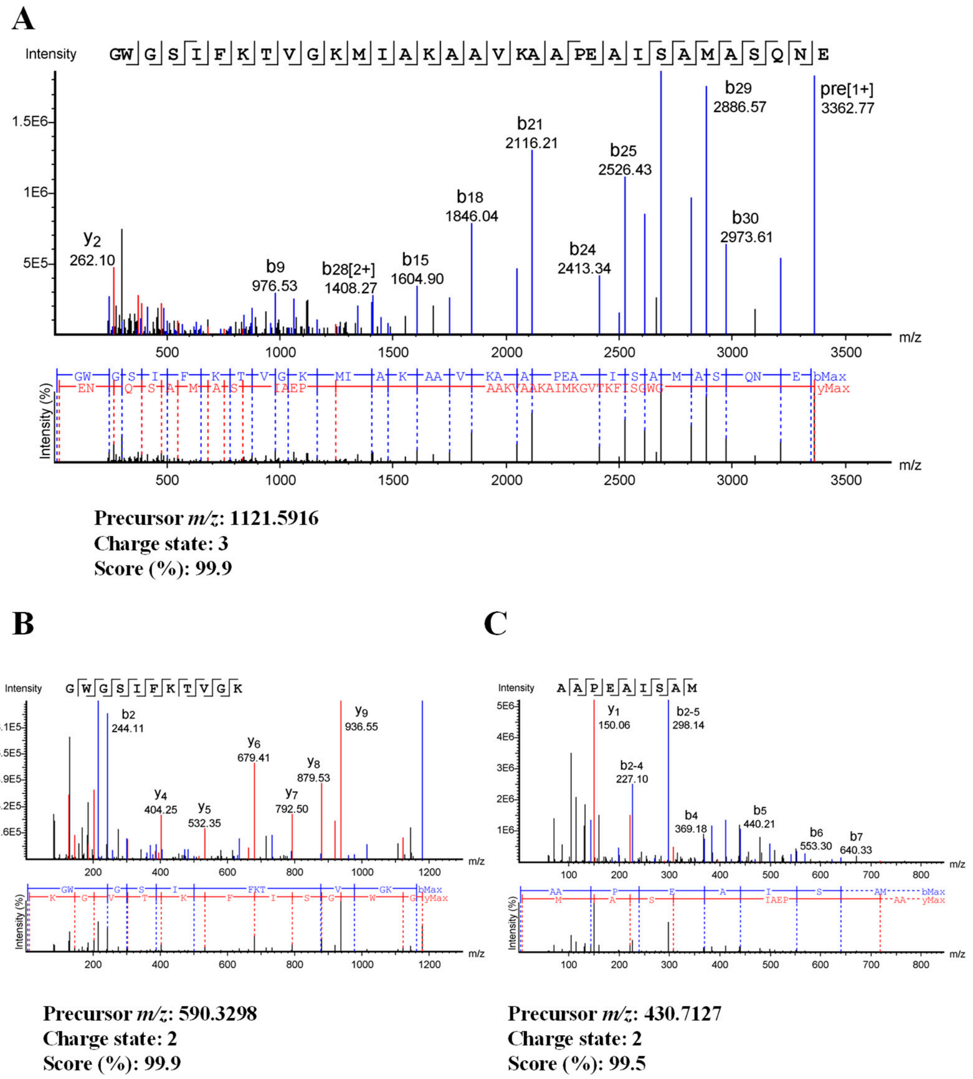

Figure S2. MS/MS spectra of pilosulin-like peptide 2. MS/MS spectra were obtained under a nonreducing condition without trypsin digestion (**A**), under a reducing condition without trypsin digestion (**B**), and under a reducing condition with trypsin digestion (**C**). The panel shows the annotated spectra and b- and y-ions of the pilosulin-like peptide 2 amino acid sequence. The peptide's precursor *m*/*z* and charge state are indicated. We referred to the transcriptome data of pilosulin-like peptide 2 for MS/MS spectrum interpretation.

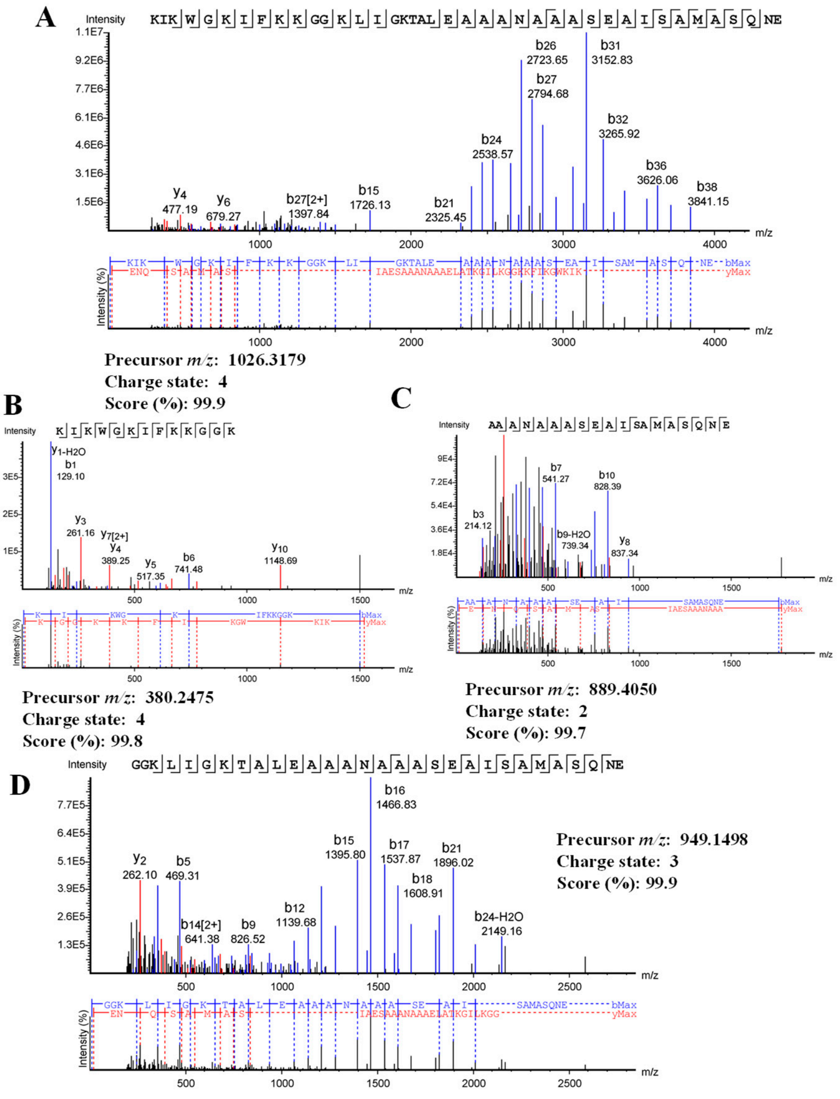

Figure S3. MS/MS spectra of pilosulin-like peptide 3. MS/MS spectra were obtained under a nonreducing condition without trypsin digestion (**A**,**D**) and under a reducing condition without trypsin digestion (**B**,**C**). The panel shows the annotated spectra and b- and y-ions of the pilosulin-like peptide 3 amino acid sequence. The peptide's precursor *m*/*z* and charge state are indicated. We referred to the transcriptome data of pilosulin-like peptide 3 for MS/MS spectrum interpretation.

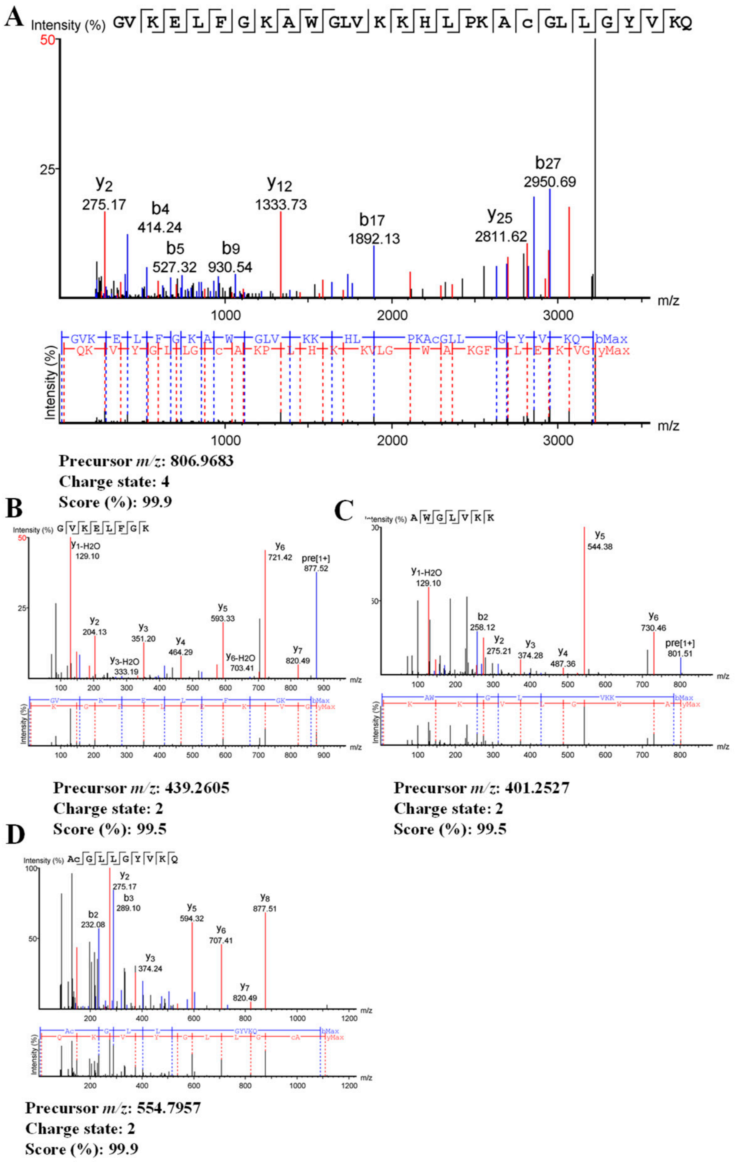

Figure S4. MS/MS spectra of pilosulin-like peptide 4. MS/MS spectra were obtained under a reducing condition without trypsin digestion (**A**) and under a reducing condition with trypsin digestion (**B**–**D**). The panel shows the annotated spectra and b- and y-ions of the pilosulin-like peptide 4 amino acid sequence. The peptide's precursor *m*/*z* and charge state are indicated. We referred to the transcriptome data of pilosulin-like peptide 4 for MS/MS spectrum interpretation.

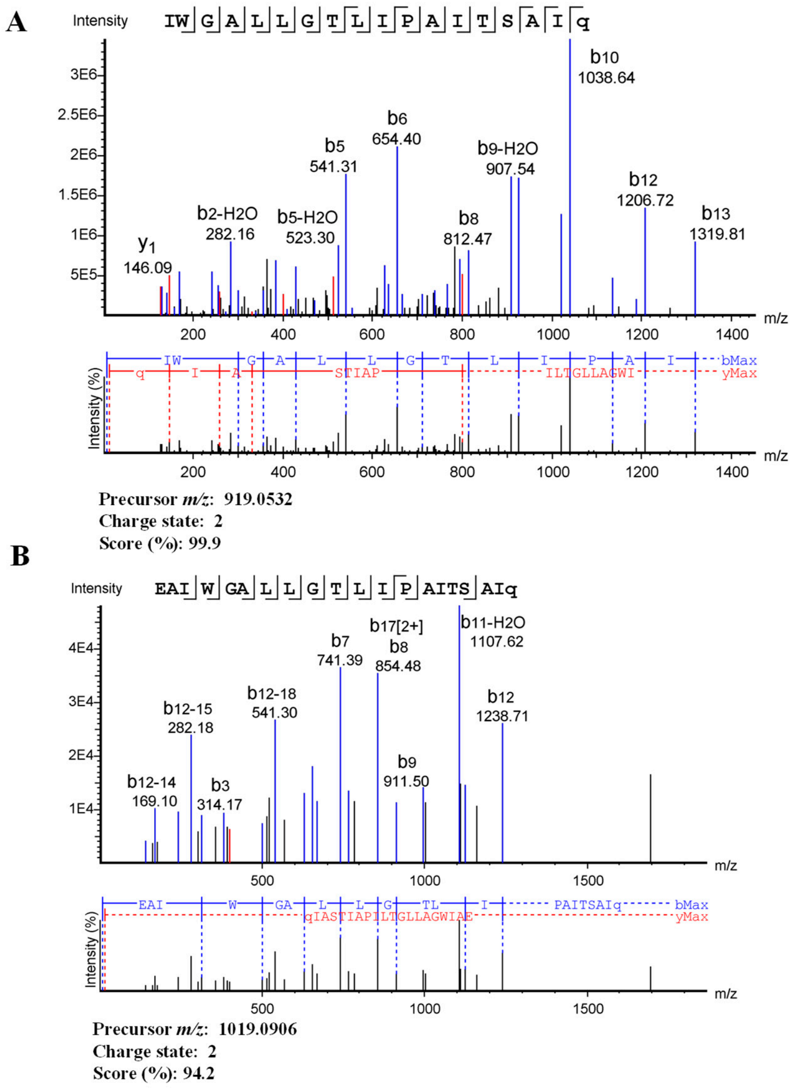

Figure S5. MS/MS spectra of pilosulin-like peptide 5. MS/MS spectra were obtained under a reducing condition without trypsin digestion (**A**,**B**). The panel shows the annotated spectra and b- and y-ions of the pilosulin-like peptide 5 amino acid sequence. The peptide's precursor *m*/*z* and charge state are indicated. We referred to the transcriptome data of pilosulin-like peptide 5 for MS/MS spectrum interpretation.

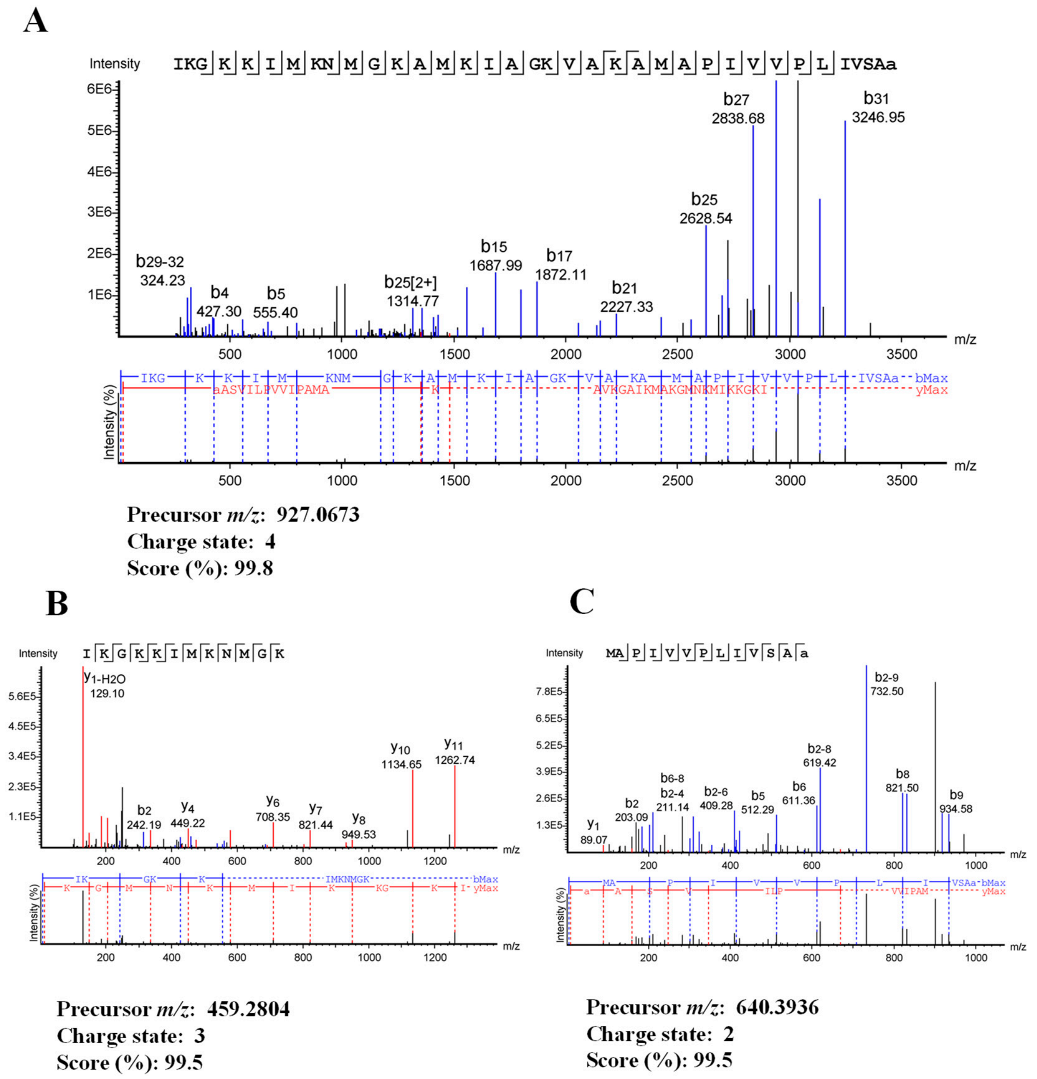

Figure S6. MS/MS spectra of pilosulin-like peptide 6. MS/MS spectra were obtained under a nonreducing condition without trypsin digestion (**A**,**C**) and under a reducing condition without trypsin digestion (**B**). The panel shows the annotated spectra and b- and y-ions of the pilosulin-like peptide 6 amino acid sequence. The peptide's precursor *m*/*z* and charge state are indicated. We referred to the transcriptome data of pilosulin-like peptide 6 for MS/MS spectrum interpretation.

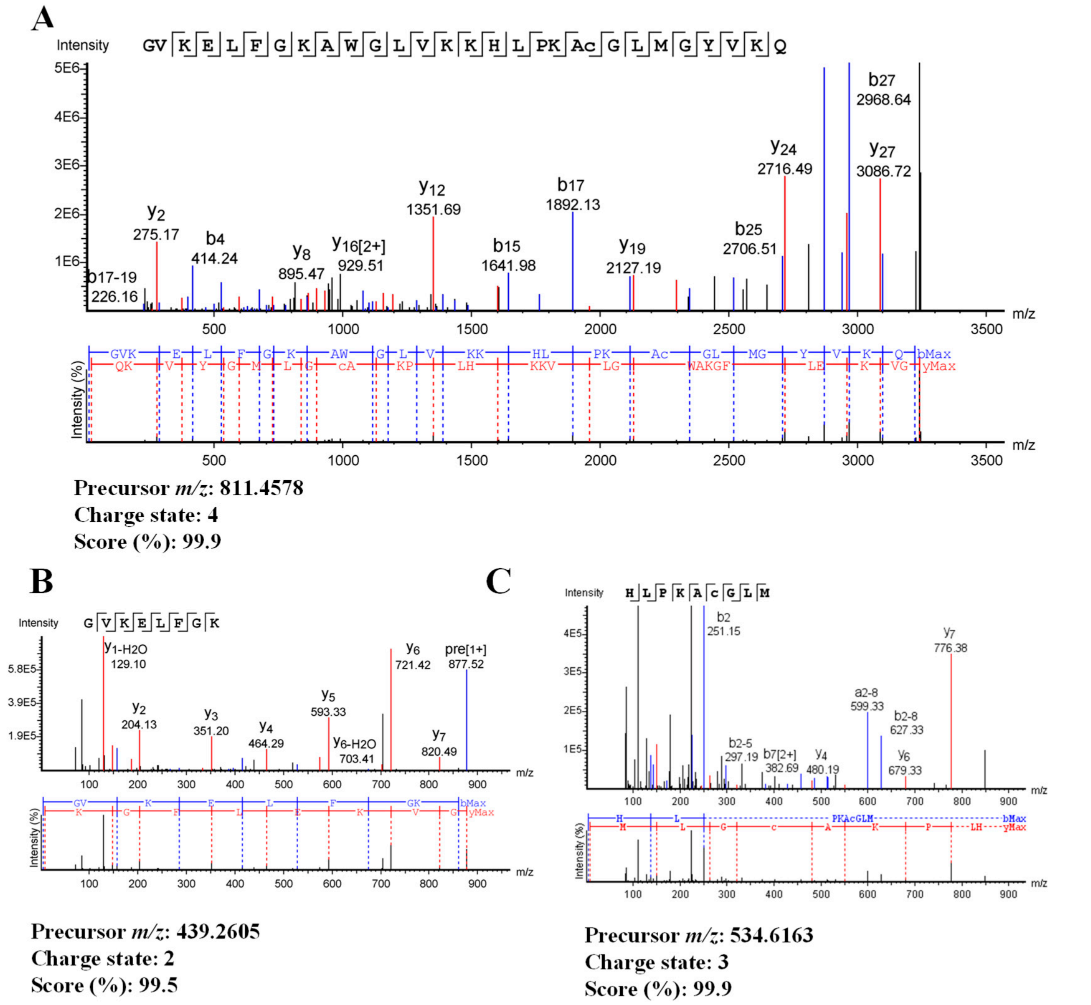

Figure S7. MS/MS spectra of pilosulin-like peptide 7. MS/MS spectra were obtained under a reducing condition without trypsin digestion (**A**,**C**) and under a reducing condition with trypsin digestion (**B**). The panel shows the annotated spectra and b- and y-ions of the pilosulin-like peptide 7 amino acid sequence. The peptide's precursor *m*/*z* and charge state are indicated. We referred to the transcriptome data of pilosulin-like peptide 7 for MS/MS spectrum interpretation.

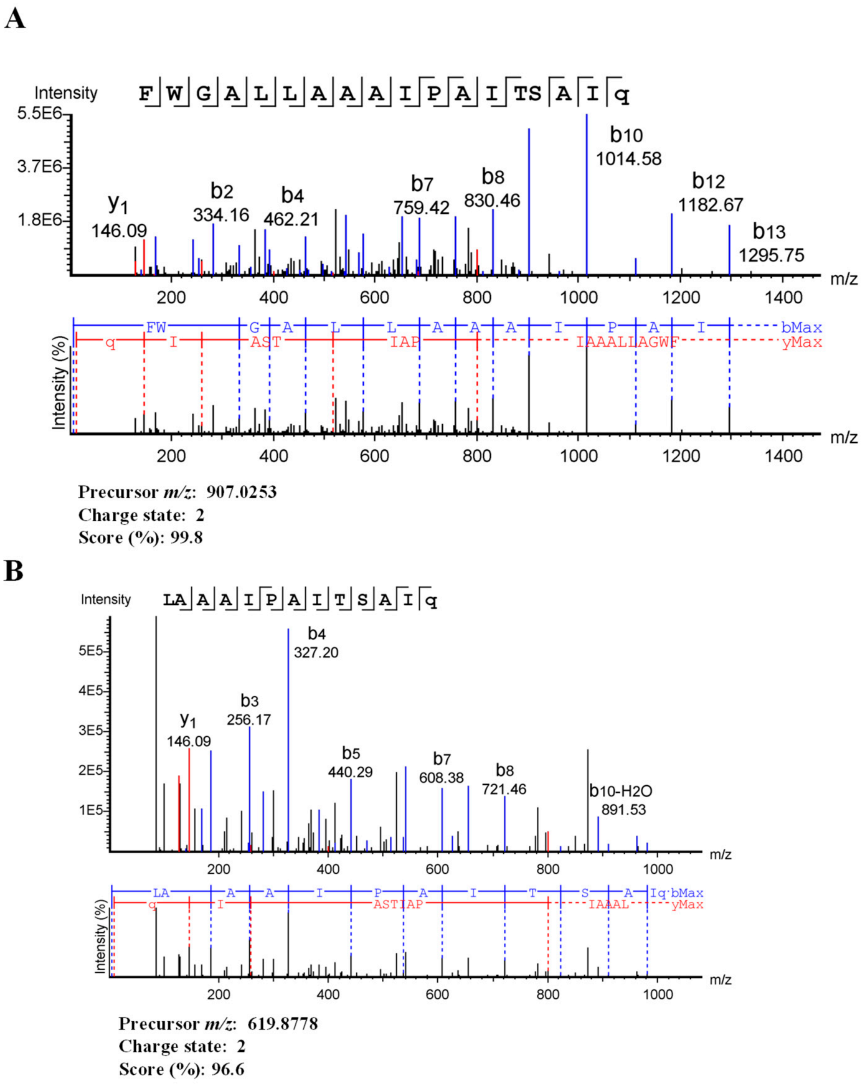

Figure S8. MS/MS spectra of pilosulin-like peptide 8. MS/MS spectra were obtained under a nonreducing condition without trypsin digestion (**A**) and under a reducing condition without trypsin digestion (**B**). The panel shows the annotated spectra and b- and y-ions of the pilosulin-like peptide 8 amino acid sequence. The peptide's precursor *m*/*z* and charge state are indicated. We referred to the transcriptome data of pilosulin-like peptide 8 for MS/MS spectrum interpretation.

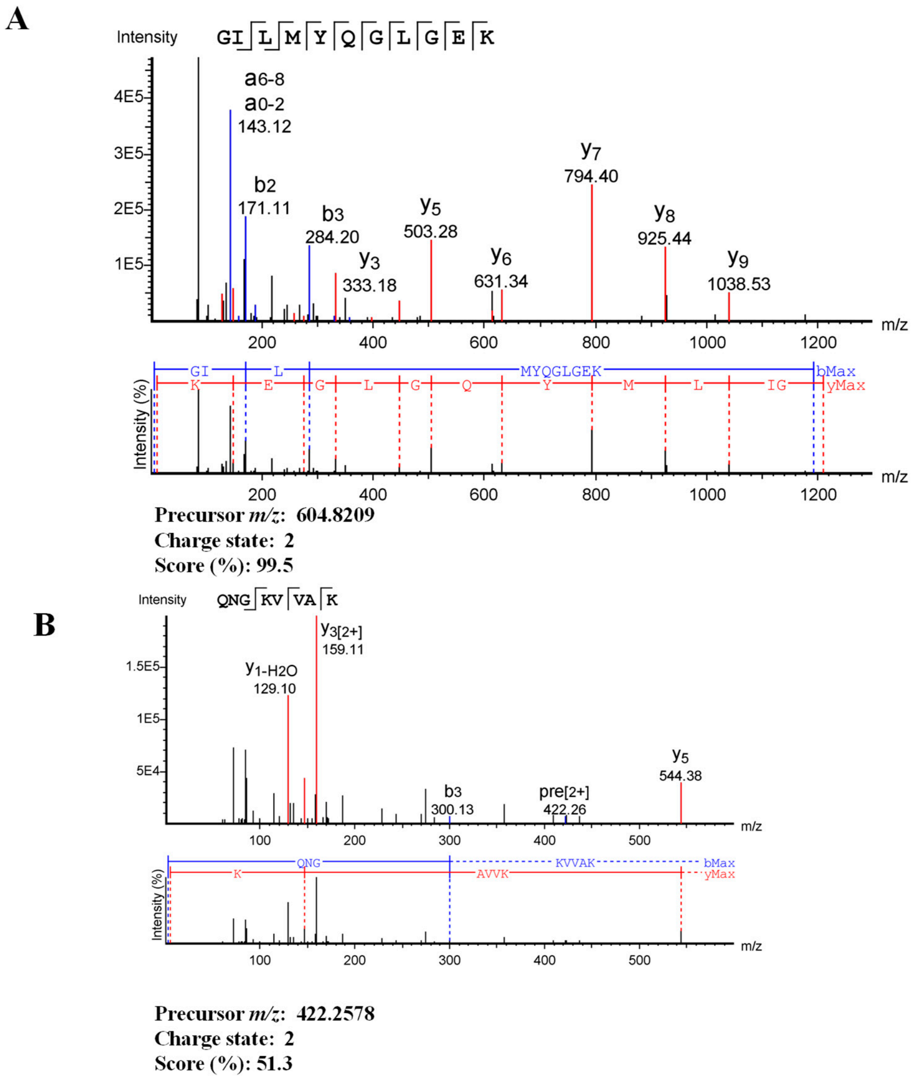

Figure S9. MS/MS spectra of pilosulin-like peptide 9. MS/MS spectra were obtained under a reducing condition with trypsin digestion (**A**,**B**). The panel shows the annotated spectra and b- and y-ions of the pilosulin-like peptide 9 amino acid sequence. The peptide's precursor *m*/*z* and charge state are indicated. We referred to the transcriptome data of pilosulin-like peptide 9 for MS/MS spectrum interpretation.

```
Met Lys Pro Ser Gly Leu Thr Leu Ala Phe Leu Val Val Phe Met   15
ATG AAA CCG TCG GGT CTC ACG TTG GCT TTC TTG GTA GTT TTT ATG   45

Met Ala Ile Met Tyr Asn Ser Val Gln Ala Glu Ala Leu Ala Asp   30
ATG GCG ATC ATG TAC AAT TCG GTA CAA GCG GAA GCA TTA GCT GAT   90
            ▼
Ala Asp Ala Glu Ala Phe Ala Glu Ala Gly Val Lys Glu Leu Phe   45
GCC GAT GCC GAA GCC TTT GCC GAG GCT GGG GTC AAG GAA TTG TTT   135

Gly Lys Ala Trp Gly Leu Val Lys Lys His Leu Pro Lys Ala Cys   60
GGT AAA GCG TGG GGA CTT GTC AAA AAG CAC TTA CCC AAA GCG TGT   180

Gly Leu Met Gly Tyr Val Lys Gln                                75
GGA CTG ATG GGA TAC GTA AAG CAA TAA TAA AGA AGA TAG ATG AAA   225

CAA CCG CAC CGA CGA TAC ACG GAA GGA CAT GAA TAA TGC TTT ACT   270
ATC AAA AAT TTC TTT CTG TTT ACA AGA ATG TCG TTT AAA TTG ATA   315
TTC TAT TAA AGA ATA AAA TTA TCT GCA AAC ACT TAA AAA AAA AAA   360
AAA AAA AAA AAA AAA AAA AAA AAA AAA AAA AAA AAA AA            398
```

Figure S10. Nucleotide and deduced amino acid sequences of pilosulin-like peptide 7. The putative mature peptide is highlighted in gray. Nucleotide sequence corresponding to the raw reads of transcriptome analysis is underlined. Arrowhead indicates the predicted N-termini of mature pilosulin-like peptide 7. The nucleotide sequence of pilosulin-like peptide 7 has been assigned DDBJ/EMBL/GenBank Accession No. LC416796.

```
TAT GTG TGA AAG CTC TTC TAT AAT AAA ATA TAA TTG TAA TAA AAC   45
CTG CAA GTA TCT CTT GTA CAA GGA ATC AAG AAA ACG TAT ATA AAC   90
GGC GCA AAT GCA AGG AAT AAA CAT CAG TTG TGC AAT AAT CAC AAC   135

                                Met Lys Leu Ser Ala Leu Ser   7
TTC AGC TTT GCT CAA TAC GAA ATG ATG AAA TTG TCG GCT TTG TCG   180

Leu Ala Phe Ala Ile Ile Leu Met Met Thr Ile Met Tyr Thr Lys   22
TTG GCT TTT GCC ATA ATC CTT ATG ATG ACG ATC ATG TAT ACT AAA   225

Ala Asp Ala Asp Ala Ser Ala Asp Ala Glu Ala Asp Ala Asp Ala   37
GCG GAT GCG GAC GCA AGT GCC GAT GCT GAG GCC GAT GCG GAT GCT   270
                ▼
Glu Ala Glu Ala Phe Trp Gly Ala Leu Leu Ala Ala Ala Ile Pro   52
GAA GCA GAA GCA TTC TGG GGT GCT CTG TTA GCA GCA GCA ATA CCA   315

Ala Ile Thr Ser Ala Ile Gln Gly Lys                           61
GCA ATA ACT TCC GCA ATA CAA GGG AAA TAA ACG AAA GTA TTG AAA   360

GCA ATC GAC GAC ACA TCA AAA TAG AAA ACG AAG AAG CGA TGA CTC   405
TGG AAC GAA AGG AAA TGA ATA ATA CTA TAA AAA ATG AAT ACT ATC   450
AAA AAT TTA TTC TAG CTT GCG TTT ATC GCA GAC AAT TGA TGT ATT   495
AAT GTC GTT CGA TTG GCA TTA CAT TGG                           522
```

Figure S11. Nucleotide and deduced amino acid sequences of pilosulin-like peptide 8. The putative mature peptide is highlighted in gray. Arrowhead indicates the predicted N-termini of mature pilosulin-like peptide 8. The nucleotide sequence of pilosulin-like peptide 8 has been assigned DDBJ/EMBL/GenBank Accession No. LC416797.

```
Met Lys Pro Ser Gly Leu Thr Phe Ala Phe Leu Val Val Phe Met   15
ATG AAA CCG TCG GGT CTC ACA TTC GCT TTC TTA GTA GTT TTT ATG   45

Met Ala Ile Met Tyr Asn Ser Val Gln Val Thr Ala Asp Ala Asp   30

ATG GCG ATC ATG TAC AAT TCG GTA CAA GTG ACA GCT GAT GCC GAT   90

Ala Asp Ala Glu Ala Glu Ala Leu Ala Asn Ala Leu Ala Glu Ala   45
GCC GAT GCT GAA GCC GAA GCC CTT GCC AAT GCC CTT GCC GAG GCT   135
▼
Gly Ile Leu Met Tyr Gln Gly Leu Gly Glu Lys Ser Asp Gly Leu   60
GGG ATC TTG ATG TAC CAA GGA CTG GGG GAA AAA AGT GAT GGA TTG   180

Asp Gln Gly Gln Asn Gly Lys Val Val Ala                       75
GAT CAA GGA CAA AAT GGG AAA GTA GTA GCA AAG AAA TAA TAA AGA   225

AGG TAG ATG AAA CAA TCG TAC CGA CAA TAC ACG GAA GGA CAT GGA   270
TAA TGC TTT ACT ATC AAA AAT TTC TTT CTA TTT ACG GCA ATG TCG   315
TTT AAA TTG ATA TTC TAT TAA AGA ATA AAA TTT TCT GCA AAC ATA   360
AAA AAA AAA AAA AAA AAA AAA AAA AAA AAA AAA                   393
```

Figure S12. Nucleotide and deduced amino acid sequences of pilosulin-like peptide 9. The putative mature peptide is highlighted in gray. Nucleotide sequence corresponding to the raw reads of transcriptome analysis is underlined. Arrowhead indicates the predicted N-termini of mature pilosulin-like peptide 9. The nucleotide sequence of pilosulin-like peptide 9 has been assigned DDBJ/EMBL/GenBank Accession No. LC416798.
